# Nationwide Impact of Centralization, Neoadjuvant Therapy, Minimally Invasive Surgery, and Standardized Pathology Reporting on R0 Resection and Overall Survival in Pancreatoduodenectomy for Pancreatic Cancer

**DOI:** 10.1245/s10434-023-13465-9

**Published:** 2023-05-20

**Authors:** Simone Augustinus, Pascale J. M. Schafrat, Boris V. Janssen, Bert A. Bonsing, Lodewijk A. A. Brosens, Olivier R. Busch, Stijn Crobach, Michail Doukas, Casper H. van Eijck, Lydia G. M. van der Geest, Bas Groot Koerkamp, Ignace H. J. T. de Hingh, G. Mihaela Raicu, Hjalmar C. van Santvoort, Marie-Louise van Velthuysen, Joanne Verheij, Marc G. Besselink, Arantza Farina Sarasqueta, for the Dutch Pancreatic Cancer Group

**Affiliations:** 1 Department of Surgery, Amsterdam UMC, University of Amsterdam, Amsterdam, The Netherlands; 2 Department of Pathology, Amsterdam UMC, University of Amsterdam, Amsterdam, The Netherlands; 3 Cancer Center Amsterdam, Amsterdam, The Netherlands; 4 Department of Surgery, Leiden University Medical Center, Leiden, The Netherlands; 5 Department of Pathology, University Medical Center Utrecht, Utrecht, The Netherlands; 6 Department of Pathology, Leiden University Medical Center, Leiden, The Netherlands; 7 Department of Pathology, Erasmus Medical Center, Erasmus University Rotterdam, Rotterdam, The Netherlands; 8 Department of Surgery, Erasmus MC Cancer Institute, Erasmus University Rotterdam, Rotterdam, The Netherlands; 9 Department of Research, Netherlands Comprehensive Cancer Organization (IKNL), Utrecht, The Netherlands; 10 Department of Surgery, Catherina Hospital, Eindhoven, The Netherlands; 11 Department of Pathology, St Antonius Hospital and Pathology DNA, Nieuwegein, The Netherlands; 12 Department of Surgery, Regional Academic Cancer Center Utrecht, University Medical Center Utrecht, Utrecht & St. Antonius Hospital Nieuwegein, Utrecht, The Netherlands

## Abstract

**Background:**

Surgeons aim for R0 resection in patients with pancreatic cancer to improve overall survival. However, it is unclear whether recent changes in pancreatic cancer care such as centralization, increased use of neoadjuvant therapy, minimally invasive surgery, and standardized pathology reporting have influenced R0 resections and whether R0 resection remains associated with overall survival.

**Methods:**

This nationwide retrospective cohort study included consecutive patients after pancreatoduodenectomy (PD) for pancreatic cancer from the Netherlands Cancer Registry and the Dutch Nationwide Pathology Database (2009–2019). R0 resection was defined as > 1 mm tumor clearance at the pancreatic, posterior, and vascular resection margins. Completeness of pathology reporting was scored on the basis of six elements: histological diagnosis, tumor origin, radicality, tumor size, extent of invasion, and lymph node examination.

**Results:**

Among 2955 patients after PD for pancreatic cancer, the R0 resection rate was 49%. The R0 resection rate decreased from 68 to 43% (2009–2019, *P* < 0.001). The extent of resections in high-volume hospitals, minimally invasive surgery, neoadjuvant therapy, and complete pathology reports all significantly increased over time. Only complete pathology reporting was independently associated with lower R0 rates (OR 0.76, 95% CI 0.69–0.83, *P* < 0.001). Higher hospital volume, neoadjuvant therapy, and minimally invasive surgery were not associated with R0. R0 resection remained independently associated with improved overall survival (HR 0.72, 95% CI 0.66–0.79, *P* < 0.001), as well as in the 214 patients after neoadjuvant treatment (HR 0.61, 95% CI 0.42–0.87, *P* = 0.007).

**Conclusions:**

The nationwide rate of R0 resections after PD for pancreatic cancer decreased over time, mostly related to more complete pathology reporting. R0 resection remained associated with overall survival.

**Supplementary Information:**

The online version contains supplementary material available at 10.1245/s10434-023-13465-9.

Surgeons aim for radical resection of pancreatic cancer during pancreatoduodenectomy (PD), defined as no microscopic residual tumor left (R0), as it is associated with improved survival.^1^ Although some discussion remains on the exact definition of R0 resection,^2^ especially for the anterior and posterior surface, both the European (Royal College of Pathologists, RCP) and the American (College of American Pathologists, CAP) definition consider R0 resection as > 1 mm tumor clearance from the margins.^3,4^

Pancreatic cancer care has evolved considerably during the past decade,^5^ including centralization,^6^ the increased use of neoadjuvant therapy,^7^ minimally invasive surgery,^8^ and the implementation of standardized pathology reporting.^9,10^ These clinical changes have been observed internationally albeit in a varying extent; a study comparing characteristics of pancreatic surgery in Germany, Sweden, the Netherlands, and the USA (2014–2017) showed a range in the use of neoadjuvant chemotherapy from 3.4 to 27.6% and for minimally invasive surgery from 4.5 to 13.5%.^11^ In addition, standardized synoptic pathology reporting has been demonstrated to improve the quality of pathology reporting in Australia, Germany, and the United Kingdom.^12–14^ These four developments have also been observed in the Netherlands specifically: (1) pancreatic surgery has been centralized from 39 to 16 hospitals,^15^ (2) the use of neoadjuvant therapy has increased from 3.8% in 1997–2012 to 8.5% in 2013–2016,^16^ (3) nationwide training programs for minimally invasive pancreatic surgery were completed in 2019,^9,10,17^ and (4) standardized synoptic pathology reporting for pancreas resection specimens was implemented from 2016 onward.^9,10^

It is unclear what the impact of these recent developments in pancreatic cancer care has been on R0 rates after PD for pancreatic cancer and whether R0 resection remains associated with improved overall survival. Therefore, the aim of this study is to investigate the impact of the four recent developments in pancreatic cancer care on the nationwide rate of R0 resection and whether R0 resection remained associated with improved overall survival.

## Patients and Methods

### Study Design

This nationwide retrospective population-based cohort study combined data from the Netherlands Cancer Registry (NCR) and the Dutch nationwide pathology database (PALGA).^18^ The NCR is a population-based registry that collects data on all newly diagnosed cancers in the Netherlands. Information is routinely extracted from the medical records by trained data managers of the NCR. PALGA is a national pathology database that registers all diagnostic pathology reports from cytology, histology, and autopsy material.

### Patient Selection

All consecutive patients undergoing PD for pancreatic cancer, including pancreatic ductal adenocarcinoma (all subtypes), and acinar cell carcinoma in the Netherlands (2009–2019), were included. In case of unclear registration and doubt on whether the patients met the inclusion criteria, consensus was reached after discussion between the first authors (S.A. and P.S.). If necessary, an experienced gastrointestinal pathologist (AFS) was consulted. When the tumor origin was not described in the pathology reports, diagnosis was based on the clinical diagnoses registered in the NCR. Patients with missing data on resection margin were excluded.

### Data Collection

All patients meeting the eligibility criteria were identified in the NCR and PALGA registry and shared, including patient identifying variables, with a trusted third party (TTP). The TTP linked the data of both registries and added a unique case number to all the matched patients and sent the dataset, including this case number, back to the NCR and PALGA. Subsequently, for all matched patients corresponding clinical variables (NCR) and pathology reports (PALGA) were selected. NCR and PALGA shared their pseudonymized databases separately with the study team together with the unique case number.

Data from the NCR included patient characteristics [i.e., age, sex, American Society Anesthesiologist (ASA) score, Charlson Comorbidity Index (CCI)], tumor characteristics [i.e., T stage and lymph node ratio (number of positive lymph nodes/total number of lymph nodes investigated, LNR)], and treatment characteristics [i.e., year of resection, surgical approach, hospital volume, and (neo)adjuvant chemotherapy/radiotherapy]. The PALGA reports included the conclusion of the pathology findings and the microscopic evaluation. From the reports, information was derived on shortest resection margin (in mm) and described radicality of the resection (R0 or R1). Moreover, it noted was whether the following items were displayed within the reports (1, yes; 0, no): histological tumor type, origin of the tumor, radicality, tumor size, extent of invasion, and lymph node examination.

### Definitions

R0 resection was defined according to both the RCP and CAP definitions as an absence of tumor cells within 1 mm of the resection margin (*>* *1 mm tumor clearance*).^3,4^ This was assessed evaluating the smallest clearance of four surgical margins and surfaces (depending on their availability): the posterior surface, pancreatic transection margin, arterial dissection surface [(superior mesenteric artery, SMA, i.e., uncinated margin or vascular margin) and venous impression surface (superior mesenteric vein, SMV, or portal vein, PV, i.e., venous groove)]. In clinical practice other definitions of R0 are also used, therefore, the* >* *1 mm tumor clearance* is compared with > *0 mm tumor clearance* (an absence of tumor cells at 0 mm of the resection margin/surface) and *>* *1 mm tumor clearance including the anterior surface*. Collection of these R0 rates from the PALGA reports was done by recoding the shortest described margin into R0 or R1 following the different definitions. When determining *>* *1 mm tumor clearance including the anterior surface*, the anterior surface was additionally taken into account. Within this definition for all margins, the 1 mm tumor clearance was used, except for the anterior surface, for which 0 mm clearance was used (as appropriate).^19^ In case the shortest margin in mm was not reported, the report was checked for descriptions of radicality. Descriptions were recoded as R0 for all definitions if stated resection margins are free, R0 resection, or complete tumor resection. In case of missing descriptions, it was coded as missing. In case of inconclusive descriptions, consensus was reached after consulting an experienced gastrointestinal pathologist (AFS).

In September 2016, standardized pathology reporting for pancreatic cancer was implemented in the Netherlands, and spread through all pancreatic surgery centers via the national pathology network PALGA (PALGA has implemented these protocols for 27 different organs).^20^ The pancreatic protocol was developed by pathologists with broad experience on pancreatic cancer pathology from four academic centers. To evaluate the influence of standardized pathology reporting on R0, the completeness of the pathology reports was used as a surrogate. To determine the completeness of pathology reports, six key elements were evaluated: histological diagnosis (i.e., histological tumor type), origin of tumor, radicality of the resection (based on descriptions or smallest margin in mm), tumor size, extent of invasion (e.g., within duodenal wall, venous patch, or no invasion), and lymph node examination (e.g., LNR or N0/N+). The description of these key elements in the reports (yes, 1; no, 0) resulted in a number between zero and six. When all six items were available, pathology reporting was considered “complete.” In analysis, a numeric variable ranging from zero to six was used. The T stage has been classified according to the Union for International Cancer Control (UICC) criteria 8th edition for patients after 2017, according to the 7th edition for patients between 2010 and 2016, and according to the 6th edition for patients in 2009.^21^ When the tumor diameter was available, it was transformed to the 8th edition, if not, the original stage was used. Centralization can be divided into four time intervals: before centralization (2009–2011: 34 centers), start of centralization (2012–2014: 24 centers), ongoing centralization (2015–2017: 18–19 centers), and after centralization (2018–2019: 17 centers).^15^ During centralization, surgical technique was not explicitly standardized. Lymphadenectomy was mostly performed according to the 2014 ISGPS definition.^22^ An artery-first approach was often used but not standardized. As multiple clinical changes happened within time, to determine the independent influence of centralization, the effect of hospital volume was evaluated. Hospital volume was divided into low and high volume, whereby high volume refers to a mean of ≥ 40 PD (for all indications) performed per year.

### Statistical Analysis

Baseline characteristics were assessed using descriptive statistics. Results were reported as proportions for categorical variables, and as mean with standard deviation (SD) or median with interquartile range (IQR) for continuous variables. Normally distributed data were compared using a Student’s t-test, categorical data using the chi-squared test, and non-normally distributed data using the Mann–Whitney *U* test. Overall survival was visualized using Kaplan–Meier curves. Median follow-up was calculated using the living patients at the end of follow-up.

Univariable logistic regression models were created to determine the increase or decrease in R0 resections and the recent developments (i.e., percentage operated in high volume hospitals, use of neoadjuvant therapy, minimally invasive surgery, and complete pathology reports) over time. A post hoc analysis was performed to evaluate the increase of the individual items described in the pathology reports over time. To identify predictors of R0 resection, relevant patient, tumor, and treatment characteristics (i.e., age, sex, neoadjuvant therapy, T stage, LNR, hospital volume, completeness of pathology reporting) were identified using univariable logistic regression models. Variables with a *P*-value < 0.20 in univariable analyses were entered in the multivariable regression models and backward step selection was used. The results were reported as odds ratio (OR) with corresponding 95% confidence interval (CI). Uni- and multivariable Cox proportional hazard models were created to determine the association between R0 resection and overall survival, adjusted for previously identified predictors, i.e., age, sex, (neo)adjuvant chemotherapy or chemoradiotherapy, T stage, LNR, hospital volume, resection year. A subgroup analysis was performed on patients that received neoadjuvant treatment (due to limited events, only variables with *P*-value < 0.20 were included in multivariate analysis). Moreover, for both predictors of R0 resection and survival analysis a sensitivity analysis was performed, including ASA and minimally invasive surgery between 2015 and 2019, as these variables were only available in the database in this period of time.

Missing data were reported but not imputed. In multivariable analysis, missing data were excluded. A *P*-value of below 0.05 was considered statistically significant. Statistical analyses were performed in RStudio version 4.0.3.

## Results

### Patient Characteristics

**Fig. 1:**
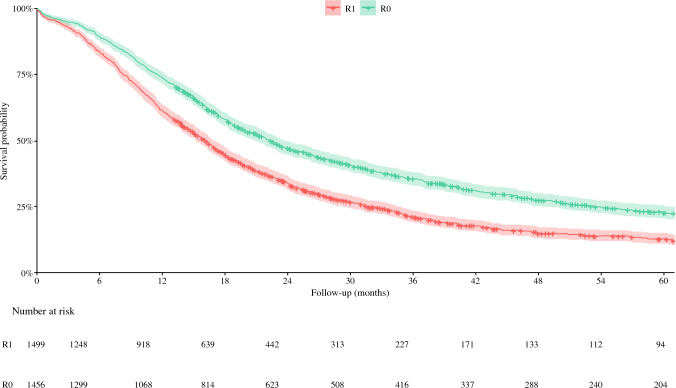
5-year-survival rate for 2955 patients after pancreatoduodenectomy for pancreatic cancer. *Using the main study definition R0: according to 1mm tumor clearance

Overall, 3025 patients were identified after PD for pancreatic cancer. After exclusion of 70 patients with missing data on R0 resection, the final cohort was made up of 2955 patients. Of these, 46.7% was female and the median age was 68.0 years (IQR 61.0–74.0, Table 1). Median overall survival was 18.5 months (95% CI 9.6–32.5, Fig. 1) with a median follow-up of patients alive at last follow-up of 38.8 months (IQR 23.4–73.2).

**Table 1:** Baseline characteristics of 2955 patients after pancreatoduodenectomy for pancreatic cancer

	All patients (*n* = 2955)
Age, median years (IQR)	68.0 (61.0–74.0)
*Missing*	0
Female	1372 (46.7%)
*Missing*	0
ASA classification	
1–2	950 (69.7%)
3–4	413 (30.3%)
*Missing*	119
CCI^a^	
0	602 (47.1%)
1	466 (36.4%)
2	156 (12.2%)
2	57 (4.4%)
*Missing*	205
Chemo(radio)therapy	
None	1273 (43.3%)
Neoadjuvant	96 (3.3%)
Adjuvant	1454 (49.5%)
Both	115 (3.9%)
*Missing*	0
Minimally invasive^a,b^	141 (9.7%)
*Missing*	28
PD performed in center with volume ≥ 40 PD/year	1361 (46.5%)
*Missing*	0
Tumor diameter in mm^c^, median (IQR)	30.0 (23.0–39.0)
*Missing*	199
T stage^d^	
1	304 (10.3%)
2	1799 (61.2%)
3	689 (23.4%)
4	149 (5.1%)
*Missing*	14
Lymph nodes evaluated (IQR)	14.0 (9.0–12.0)
*Missing*	24
Lymph nodes positive (IQR)	2.0 (0.0–4.0)
*Missing*	21
LNR, median (IQR)	0.1 (0.0-0.3)
*Missing*	46
Year of resection	
2009–2011	624 (21.1%)
2012–2013	573 (19.5%)
2014–2015	549 (18.7%)
2016–2017	604 (20.6%)
2018–2019	588 (20.1%)
*Missing*	0

Numbers are depicted as numbers with valid percentages, unless indicated otherwise. *ASA* American Society of Anesthesiologists, *CCI* Carlson Comorbidity Index, *PD* pancreatoduodenectomy, *LNR* lymph node ratio, *SD* standard deviation, *IQR* interquartile range

^a^Only in patients between 2015 and 2019 (*n* = 1482)

^b^including patients with conversion to open surgery (*n* = 34)

^c^based on PALGA reports

^d^if pT stage was unavailable, cT stage was used

### R0 Resection

**Fig. 2:**
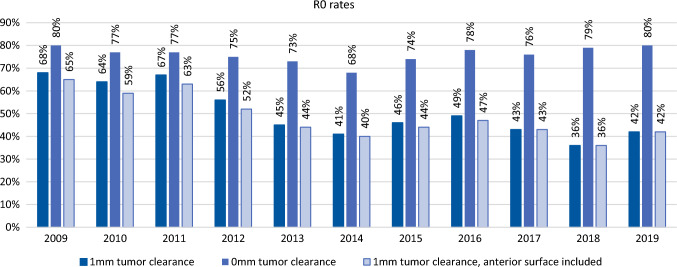
Annual R0 resection rates after pancreatoduodenectomy for pancreatic cancer, according to three most commonly used definitions for R0 resection. *Main study definition R0: according to 1 mm tumor clearance. Other used definitions: 0 mm tumor clearance and 1 mm tumor clearance, anterior surface included

The overall rate of R0 resection (*>* *1 mm clearance*) was 49.3% and decreased over time from 67.5% in 2009 to 42.6% in 2019 (OR 0.89, 95% CI 0.87–0.91, *P* < 0.001, Fig. 2).

### Recent Developments

The percentage of resections performed in high-volume centers (≥ 40 per year) increased from 35.5% in 2009 to 67.5% in 2019 (*P* < 0.001). The use of neoadjuvant therapy increased from 0% in 2009 to 28.4% in 2019 (*P* < 0.001). The use of minimally invasive PD increased from 0% in 2015 up to 19.4% in 2019 (*P* < 0.001). Complete pathology reporting (i.e., all six items were described in the pathology report) increased from 39.0% in 2009 to 81.5% in 2019 (*P* < 0.001). Nearly all pathology reports described the histological diagnosis (98.7%), lymph node examination (96.9%), tumor size (93.2%), and radicality of the resection (100%, but 70 patients excluded from analysis as only patients with a description of radicality were included). The description origin of the tumor fluctuated between 54.7 and 86.2% over time, and the extent of invasion between 60.9 and 91.1% (Supplementary Fig. 1). Evaluating the individual items described within the pathology reports, the reporting of all items increased over time, except for the extent of invasion (*P* = 0.069).

### Association of Recent Developments with R0 Resection

Surgery in high-volume centers (Table 2), use of neoadjuvant chemotherapy (Table 2), and minimally invasive surgery (Supplementary Table 1) were not associated with R0 resection. Completeness of pathology reporting (i.e., number between zero and six based on items described in the pathology reports) was independently associated with R0 resection, together with age, T stage, and LNR (Table 2).

**Table 2:** Predictors for R0 resection in 2955 patients after pancreatoduodenectomy for pancreatic cancer

	According to 1 mm tumor clearance (main study definition)			
	Univariable analysis	*P*-value	Multivariable analysis^a^	*P*-value
	OR (95% CI)		OR (95% CI)	
Age	0.99 (0.98–0.99)	**0.003**	0.99 (0.98–0.99)	**0.008**
Female	1.04 (0.89–1.20)	0.604		
Neoadjuvant chemo(radio)therapy	1.29 (0.98–1.71)	**0.075**		
T stage^b^				
1	Reference		Reference	
2	0.37 (0.27–0.48)	**< 0.001**	0.49 (0.37–0.64)	**< 0.001**
3	0.36 (0.28–0.47)	**< 0.001**	0.35 (0.26–0.47)	**< 0.001**
4	0.19 (0.13–0.29)	**< 0.001**	0.23 (0.14–0.35)	**< 0.001**
LNR	0.22 (0.15–0.31)	**< 0.001**	0.29 (0.20–0.42)	**< 0.001**
PD performed in high-volume center (≥ 40 PD/year)	0.87 (0.76–1.01)	**0.077**		
Completeness of pathology report^c^	0.77 (0.70–0.84)	**< 0.001**	0.76 (0.69–0.83)	**< 0.001**

Bold numbers in univariable analysis indicate variables that were entered in multivariable analysis. Bold numbers in multivariable analysis indicate statistical significance (*P* < 0.05). *OR* odds ratio, *LNR* lymph node ratio, *PD* pancreatoduodenectomy

^a^Multivariable analysis after backward step selection in 2876 patients (62 deleted due to missing values)

^b^if pT stage was unavailable, cT stage was used

^c^score between 0–6 based on the following variables: histological diagnosis, origin of the tumor, resection margin, extent of invasion, LNR, and tumor size

### Overall Survival

R0 resection was associated with improved overall survival (HR 0.72, 95% CI 0.66–0.79, *P* < 0.001, Table 3) after adjustment for previously identified predictors (including year of resection). In the subgroup of 214 patients who received neoadjuvant treatment, R0 resection remained significantly associated with overall survival (HR 0.61, 95% CI 0.47–0.97, *P* = 0.007, Table 4). In both sensitivity analysis, correcting for ASA and minimally invasive surgery (2015–2019, Supplementary Table 2), and excluding the 27% of patients in which the margins were based on descriptive text, R0 resection remained associated with overall survival.

**Table 3:** Predictors for overall survival in 2955 patients after pancreatoduodenectomy for pancreatic cancer

	According to 1 mm tumor clearance (main study definition)			
	Univariable analysis	*P*-value	Multivariable analysis^a^	*P*-value
	HR (95% CI)		HR (95% CI)	
R0 resection^b^	0.68 (0.63–0.74)	**< 0.001**	0.72 (0.66–0.79)	**< 0.001**
Age	1.01 (1.01–1.02)	**< 0.001**	1.00 (1.00–1.01)	**0.047**
Female	0.98 (0.90–1.06)	**0.548**		
Chemo(radio)therapy				
None	Reference		Reference	
Neoadjuvant	0.81 (0.62–1.05)	**0.104**	1.03 (0.79–1.35)	0.81
Adjuvant	0.67 (0.61–0.73)	**< 0.001**	0.61 (0.56–0.67)	**< 0.001**
Both	0.52 (0.41–0.67)	**< 0.001**	0.73 (0.57–0.95)	**0.017**
T stage^c^				
1	Reference		Reference	
2	1.76 (1.50–2.07)	**< 0.001**	1.52 (1.29–1.79)	**< 0.001**
3	2.24 (1.89–2.66)	**< 0.001**	1.82 (1.52–2.17)	**< 0.001**
4	2.35 (1.87–2.95)	**< 0.001**	1.59 (1.25–2.01)	**< 0.001**
LNR	4.66 (3.93–5.54)	**< 0.001**	4.09 (3.41–4.90)	**< 0.001**
PD performed in center with volume ≥ 40 PD/year	0.81 (0.74–0.87)	**< 0.001**	0.84 (0.77–0.91)	**< 0.001**
Year of resection	0.98 (0.63–0.74)	**0.006**		

Bold numbers in univariable analysis indicates variables that were entered in multivariable analysis. Bold numbers in multivariable analysis indicates statistical significance (*P* < 0.05). OR: Odds ratio. LNR: lymph node ratio. PD: pancreatoduodenectomy

^a^Multivariable analysis after backward step selection in 2898 patients (57 deleted due to missing values

^b^Following the definitions above/on top of the table

^c^If pT stage was unavailable, cT stage was used

**Table 4:** Predictors for overall survival in the subgroup of 214 patients with pancreatic cancer after neoadjuvant treatment

	According to 1 mm tumor clearance (main study definition)			
	Univariable analysis	*P*-value	Multivariable analysis^a^	*P*-value
	HR (95% CI)		HR (95% CI)	
R0 resection^b^	0.52 (0.36–0.74)	**< 0.001**	0.61 (0.42–0.87)	**0.007**
Age	1.02 (1–1.04)	0.124		
Female	0.87 (0.61–1.23)	0.43		
Adjuvant chemotherapy	0.65 (0.46–0.93)	**0.018**	0.68 (0.47–0.97)	**0.033**
ASA				
1–2	Reference			
3–4	0.99 (0.62–1.57)	0.953		
Minimally invasive	1.36 (0.75–2.47)	0.308		
T stage^c^				
1	Reference			
2	1.22 (0.78–1.90)	**0.378**		
3	2.14 (1.10–4.18)	**0.025**		
4	2.43 (0.93–6.35)	**0.069**		
LNR	8.99 (3.33–24.29)	**< 0.001**	8.29 (2.93–23.47)	**< 0.001**
PD performed in center with volume ≥ 40 PD/year	1.06 (0.68–1.64)	0.8		
Year of resection	1 (0.91–1.09)	0.944		

Bold numbers in univariable analysis indicate variables that were entered in multivariable analysis (*P* < 0.20). Bold numbers in multivariable analysis indicate statistical significance (*P* < 0.05). *OR* odds ratio, *LNR* lymph node ratio, *PD* pancreatoduodenectomy

^a^Multivariable analysis after backward step selection in 213 patients (1 deleted due to missing values)

^b^following the definitions above/on top of the table

^c^if pT stage was unavailable, cT stage was used

### Other Common Definitions for R0

The rate of *R0 resections* *0 mm clearance* was 76.1% and did not significantly change over time (OR 1.01, 95% CI 0.99–1.04, *P* = 0.369, Fig. 2). The rate of *R0 resections > 1 mm clearance including the anterior surface* was 47.5% and significantly decreased over time from 64.5 to 42.2% (OR 0.91, 95% CI 0.89–0.93, *P* < 0.001). The influence of the recent developments was mostly the same using all definitions, however, more complete pathology reporting was not associated with *R0 resection > 0 mm tumor clearance* definition, and hospital volume was associated with *R0 resection > 1 mm clearance including the anterior surface* definition (Supplementary Table 3). R0 resection was associated with survival using all definitions (Supplementary Table 4). However, in the subgroup of patients who received neoadjuvant therapy, this was the not the case using the *R0 resection > 0 mm tumor clearance* definition (HR 0.84, 95% CI 0.53–1.34, *P* = 0.473, Supplementary Table 5).

## Discussion

This first nationwide analysis evaluating the influence of four recent developments in pancreatic cancer care in the last decade on R0 resections, found a decreasing rate of R0 resections (from 67.5 to 42.6%), mostly related to the completeness of pathology reporting. Hospital volume, neoadjuvant therapy, and minimally invasive PD were not associated with R0 resection. R0 remained associated with overall survival, as well as in the subgroup of patients who received neoadjuvant therapy.

Previous studies have reported a relationship between R0 resection and overall survival using different definitions for R0 resection.^23,24^ However, these studies did not include trends in R0 resection and associations with accompanying clinical changes, even though these clinical changes are apparent worldwide.^11,12,25^ This study confirms that an R0 resection is related to the completeness of pathology reporting. This is not surprising, as reporting of key pathology items can be interpreted as a sign of quality, and standardized pathology reporting is associated with decreased R0 resection rates.^12–14,26^ It can therefore be hypothesized that the implementation of synoptic reporting based on nationwide protocols in the Netherlands has resulted in more complete pathology reports (due to a tendency toward a more accurate examination of PD specimen), resulting in the association with decreased R0 rates.

The present study did not find an association of centralization with the rate of R0 resections, even though other studies have suggested such an assocation.^27,28^ The main hypothesis within these studies is that high-volume centers have more specialized surgeons, increasing R0 resections. However, one could also hypothesize that more accurate and extensive assessment of margins by specialized pathologists in high-volume centers could lead to a decrease in R0 resections. A previous study showed that pathology reports of low-volume hospitals lacked more data (25% versus 15%, *P* < 0.001).^22^ This hypothesis can be substantiated by Supplementary Fig. 1. In the final period (2018–2019: centralization complete), individual items evaluated within complete pathology reporting are highest. These contradicting effects of centralization (on the one hand more specialized surgeons, but also more specialized pathologists) could dilute the effect of hospital volume in multivariable analysis. The present study found no association of minimally invasive surgery with the rate of R0 resections. A meta-analysis of robotic versus open PD, including pooled data from eight studies, showed an increased R0 resection rate with robotic PD (OR 0.40, 95% CI 0.20–0.77, *P* = 0.006).^29^ However, no randomized trials have compared robotic with open PD. Four randomized trials comparing laparoscopic and open pancreatoduodenectomy found no difference in R0 resections.^30–33^

In contrast with recent data in which neoadjuvant chemoradiotherapy improved R0 resection rates,^34^ in the present study we could not confirm neoadjuvant therapy as a predictor for R0 resection. This apparently contradictory result could be at least partly explained by the fact that assessment of margins after neoadjuvant therapy is known to be challenging, due to effect of the therapy on the tumor bed (giving the potential to cause the distance between the rest tumor cells being larger than 1 mm) and a chance of overreporting of R0 due to subtotal sampling in more advanced tumors.^35^ This highlights the importance of a thorough pathological evaluation, in addition to the surgical resection and oncological treatment, especially in patients after neoadjuvant therapy.

The results of this study should be interpreted in light of several limitations. First, heterogeneity exists in specimen sampling and interpretation of definitions of the different margins and surfaces among the different centers, which is known to influence the R0 rate. However, this reflects clinical practice, and lack of consensus regarding definitions of margins and surfaces is also apparent at the international level.^2^ Furthermore, when using different definitions of R0 resection, the predictors for R0 resections changed. This highlights the urgent need for international and multidisciplinary consensus on details of the R0 resection definition, especially on the anterior and posterior surface. Second, data on postoperative complications and vascular resections were not available in our database. Additionally, data of minimally invasive surgery and ASA score were only available from 2015 onward, however, sensitivity analysis showed that this did not influence the results. Third, the R0 resection rates in this study were low (49.3%), especially compared with studies (mainly in high-volume centers) in the USA, indicating an R0 rate up to 85%.^27,36^ However, these differences may be explained to a large extent by difference in definitions used in clinical practices and margins assessed, which, due to the International Collaboration on Cancer Reporting (ICCR) consensus article, will hopefully become more similar in the future,^19^ increasing the generalizability of the present article.

The main strength of this nationwide study is that it provides a unique overview of a decade of four developments in PD for pancreatic cancer including all aspects regarding R0 resection rates (i.e., different definitions used and clinical developments) and its impact on overall survival.

The nationwide R0 resection rate in PD for pancreatic cancer significantly decreased over the course of a decade, mostly related to completeness of pathology reporting. An R0 resection remains associated with improved overall survival. The increasing centralization, use of neoadjuvant therapy, and minimally invasive surgery were not related to the rate of R0 resections. Our results emphasize the importance of the quality and reporting of pathological evaluation of pancreas resection specimens and a uniform definition of R0 resection.

## Supplementary Information

Below is the link to the electronic supplementary material.

Supplementary file1 (DOCX 41 kb)
