# Supplementary material for: Nationwide Impact of Centralization, Neoadjuvant Therapy, Minimally Invasive Surgery, and Standardized Pathology Reporting on R0 Resection and Overall Survival in Pancreatoduodenectomy for Pancreatic Cancer
Source: Ann Surg Oncol. 2023 May 20;30(8):5051–60. doi: 10.1245/s10434-023-13465-9 (PMC10319672; doi:10.1245/s10434-023-13465-9)
Supplement: Supplementary file 1 — Supplementary file1 (DOCX 41 kb) [file 10434_2023_13465_MOESM1_ESM.docx]

**Supplementary Table 1.** Predictors for R0 resection between 2015-2019 (including ASA and minimally invasive surgery)

|  | **According to 1mm tumor clearance**  **(main study definition)** | | | | **According to 0mm tumor clearance** | | | | **According to 1mm tumor clearance,  including anterior surface** | | | |
| --- | --- | --- | --- | --- | --- | --- | --- | --- | --- | --- | --- | --- |
|  | Univariable analysis  OR (95%CI) | P-value | Multivariable analysis^a^ OR (95% CI) | P-value | Univariable analysis  OR (95%CI) | P-value | Multivariable analysis^b^ OR (95% CI) | P-value | Univariable analysis  OR (95%CI) | P-value | Multivariable analysis^c^ OR (95% CI) | P-value |
| Age | 0.98 (0.97-0.99) | **0.005** | 0.98 (0.98-0.99) | **0.014** | 0.97 (0.96-0.99) | **<0.001** | 0.97 (0.96-0.99) | **0.001** | 0.99 (0.98-0.99) | **0.012** | 0.98(0.98-0.99) | **0.033** |
| Female | 1.02 (0.83-1.25) | 0.858 |  |  | 0.96 (0.75-1.22) | 0.718 |  |  | 0.98 (0.79-1.21) | 0.859 |  |  |
| Neoadjuvant chemo(radio)therapy | 1.66 (1.22-2.26) | **0.001** |  |  | 1.51 (1.02-2.28) | **0.045** |  |  | 1.67 (1.23-2.28) | **0.003** |  |  |
| ASA ≥ 3 | 0.74 (0.58-0.93) | **0.011** |  |  | 0.86 (0.66-1.13) | 0.277 |  |  | 0.76 (0.59-0.96) | **0.022** |  |  |
| Minimally invasive surgery | 0.99 (0.69-1.41) | 0.975 |  |  | 1.21 (0.79-1.89) | 0.493 |  |  | 1.02 (0.72-1.45) | 0.901 |  |  |
| T stage^d^  1  2  3  4 | reference 0.39 (0.28-0.53) 0.30 (0.19-0.45) 0.34 (0.19-0.61) | **<0.001 <0.001 <0.001** | reference 0.48 (0.35-0.67) 0.48 (0.35-0.67) 0.46 (0.24-0.84) | **<0.001 <0.001 0.012** | reference 0.51 (0.31-0.79) 0.29 (0.17-0.49) 0.18 (0.09-0.35) | **0.013 <0.001 <0.001** | reference 0.62 (0.38-0.98) 0.38 (0.22-0.65) 0.26 (0.12-0.41) | **0.050 <0.001 <0.001** | reference 0.38 (0.27-0.52) 0.29 (0.19-0.43) 0.32 (0.18-0.57) | **<0.001 <0.001 <0.001** | reference 0.47(0.34-0.66) 0.40(0.26-0.62) 0.43(0.23-0.80) | **<0.001 <0.001 0.008** |
| LNR | 0.10 (0.06-1.18) | **<0.001** | 0.14 (0.07-0.25) | **<0.001** | 0.16 (0.09-0.29) | **<0.001** | 0.22 (0.12-0.41) | **<0.001** | 0.09 (0.05-0.17) | **<0.001** | 0.13(0.70-0.24) | **<0.001** |
| PD performed in center with volume ≥40 PD/year | 0.99 (0.81-1.22) | 0.954 |  |  | 1.11 (0.87-1.42) | 0.400 |  |  | 0.95 (0.78-1.17) | 0.661 |  |  |
| Completeness of pathology report^e^ | 0.77 (0.68-0.87) | **<0.001** | 0.75 (0.65-0.86) | **<0.001** | 1.08 (0.93-1.25) | 0.289 |  |  | 0.78 (0.69-0.89) | **<0.001** | 0.76(0.66-0.88) | **<0.001** |

Bold numbers in univariable analysis indicates variables that were entered in multivariable analysis. Bold numbers in multivariable analysis indicates statistical significance (p<0.05). ASA: American Society of Anesthesiologists. OR: Odds ratio. LNR: lymph node ratio. PD: pancreatoduodenectomy. ^a,b^Multivariable analysis after backward step selection in 1484 patients (11 deleted due to missing values). ^c^Multivariable analysis after backward step selection in 1481 patients (14 deleted due to missing values). ^d^If pT stage was unavailable, cT stage was used.  ^e^Score between 0-6 based on the following variables: histological diagnosis, origin of the tumor, resection margin, extent of invasion, LNR, tumor size.

|  | **According to 1mm tumor clearance**  **(main study definition)** | | | | **According to 0mm tumor clearance** | | | | **According to 1mm tumor clearance,  including anterior surface** | | | |
| --- | --- | --- | --- | --- | --- | --- | --- | --- | --- | --- | --- | --- |
|  | Univariable analysis   HR (95%CI) | P-value | Multivariable analysis^a^  HR (95%CI) | P-value | Univariable analysis   HR (95%CI) | P-value | Multivariable analysis^b^  HR (95%CI) | P-value | Univariable analysis   HR (95%CI) | P-value | Multivariable analysis^c^  HR (95%CI) | P-value |
| R0 resection^d^ | 0.68 (0.60-0.77) | **<0.001** | 0.76 (0.66-0.88) | **<0.001** | 0.62 (0.54-0.72) | **<0.001** | 0.72 (0.62-0.84) | **<0.001** | 0.67 (0.59-0.76) | **<0.001** | 0.75 (0.65-0.87) | **<0.001** |
| Age | 1.02 (1.01-1.02) | **<0.001** |  |  | Same as 1mm tumor clearance | |  |  | Same as 1mm tumor clearance | |  |  |
| Female | 0.99 (0.88-1.12) | 0.872 |  |  |  |  |  |  |  |  |  |  |
| Chemo(radio)therapy  None  Neo-adjuvant  Adjuvant  Both | reference 0.74 (0.55-0.98) 0.59 (0.52-0.67) 0.51 (0.38-0.67) | **0.038 <0.001 <0.001** | reference 0.79 (0.58-1.09) 0.47 (0.41-0.54) 0.61 (0.45-0.83) | 0.152 **<0.001 0.001** |  |  | reference 0.81 (0.59-1.11) 0.48 (0.42-0.55) 0.59 (0.44-0.80) | 0.193 **<0.001 <0.001** |  |  | reference 0.79 (0.65-1.09) 0.47 (0.41-0.54) 0.61 (0.45-0.83) | 0.151 **<0.001 0.001** |
| ASA ≥ 3 | 1.3 (1.14-1.50) | **<0.001** | 1.19 (1.03-1.37) | **0.017** |  |  | 1.20 (1.04-1.38) | **0.012** |  |  | 1.19 (1.03-1.37) | **0.016** |
| Minimally invasive surgery | 1.08 (0.86-1.34) | **0.517** |  |  |  |  |  |  |  |  |  |  |
| T stage^e^  1  2  3  4 | reference 1.71 (1.37-2.14) 2.35 (1.81-3.04) 2.03 (1.44-2.87) | **<0.001 <0.001 <0.001** | reference 1.19 (1.03-1.37) 1.49 (1.17-1.89) 1.78 (0.87-1.93) | **0.001 <0.001** 0.194 |  |  | reference 1.53 (1.20-1.95) 1.77 (1.33-2.35) 1.26 (0.85-1.88) | **<0.001 <0.001** 0.246 |  |  | reference 1.48 (1.16-1.89) 1.78 (1.34-2.36) 1.29 (0.87-1.91) | **0.001 <0.001** 0.211 |
| LNR | 6.19 (4.68-8.19) | **<0.001** | 5.59 (4.08-7.66) | **<0.001** |  |  | 5.63 (4.12-7.69) | **<0.001** |  |  | 5.56 (4.06-7.62) | **<0.001** |
| PD performed in center with volume ≥40 PD/year | 0.87 (0.77-0.98) | **0.023** |  |  |  |  |  |  |  |  |  |  |

**Supplementary Table 2.** Predictors for overall survival between 2015-2019 (including ASA and minimally invasive surgery)

Bold numbers in univariable analysis indicates variables that were entered in multivariable analysis. Bold numbers in multivariable analysis indicates statistical significance (p<0.05). OR: Odds ratio. LNR: lymph node ratio. PD: pancreatoduodenectomy. ^a^Multivariable analysis after backward step selection in 1488 patients (7 deleted due to missing values). ^b^Multivariable analysis after backward step selection in 1366 patients (129 deleted due to missing values). ^c^Multivariable analysis after backward step selection in 1368 patients (158 deleted due to missing values). ^d^Following the definitions above / on top of the table. ^e^If pT stage was unavailable, cT stage was used.

**Supplementary Table 3.** Predictors for R0 resection in 2955 patients after pancreatoduodenectomy for pancreatic cancer using different definitions

Bold numbers in univariable analysis indicates variables that were entered in multivariable analysis. Bold numbers in multivariable analysis indicates statistical significance (p<0.05). OR: Odds ratio. LNR: lymph node ratio. PD: pancreatoduodenectomy. ^a^Multivariable analysis after backward step selection in 2876 patients (62 deleted due to missing values). ^b^Multivariable analysis after backward step selection in 2881 patients (57 deleted due to missing values). ^c^Multivariable analysis after backward step selection in 2874 patients (64 deleted due to missing values). ^d^If pT stage was unavailable, cT stage was used. ^e^Score between 0-6 based on the following variables: histological diagnosis, origin of the tumor, resection margin, extent of invasion, LNR, tumor size.

|  | **According to 1mm tumor clearance**  **(main study definition)** | | | | **According to 0mm tumor clearance** | | | | **According to 1mm tumor clearance,  including anterior surface** | | | |
| --- | --- | --- | --- | --- | --- | --- | --- | --- | --- | --- | --- | --- |
|  | Univariable analysis  OR (95%CI) | P-value | Multivariable analysis^a^ OR (95% CI) | P-value | Univariable analysis  OR (95%CI) | P-value | Multivariable analysis^b^ OR (95% CI) | P-value | Univariable analysis  OR (95%CI) | P-value | Multivariable analysis^c^ OR (95% CI) | P-value |
| Age | 0.99 (0.98-0.99) | **0.003** | 0.99 (0.98-0.99) | **0.008** | 0.98 (0.97-0.99) | **0.007** | 0.98 (0.97-0.99) | **0.001** | 0.99 (0.98-0.99) | **0.008** | 0.99 (0.98-0.99) | **0.019** |
| Female | 1.04 (0.89-1.20) | 0.604 |  |  | 1.04 (0.87-1.23) | 0.685 |  |  | 1.06 (0.92-1.23) | 0.425 |  |  |
| Neoadjuvant chemo(radio)therapy | 1.29 (0.98-1.71) | **0.075** |  |  | 1.60 (1.12-2.35) | **0.012** |  |  | 1.36 (1.03-1.80) | **0.031** |  |  |
| T stage^d^  1  2  3  4 | reference 0.39 (0.31-0.52) 0.28 (0.21-0.37) 0.19 (0.13-0.29) | **<0.001 <0.001 <0.001** | reference 0.49 (0.37-0.64) 0.35 (0.26-0.47) 0.23 (0.14-0.35) | **<0.001 <0.001 <0.001** | reference 0.44 (0.29-0.64) 0.26 (0.17-0.39) 0.11 (0.07-0.17) | **<0.001 <0.001**  **<0.001** | reference 0.51 (0.34-0.74)  0.32 (0.21-0.48) 0.13 (0.08-0.22) | **<0.001 <0.001 <0.001** | reference 0.39 (0.29-0.49) 0.27 (0.20-0.36) 0.17 (0.11-0.26) | **<0.001 <0.001 <0.001** | reference 0.46 (0.35-0.61) 0.33 (0.24-0.44) 0.19 (0.13-0.31) | **<0.001 <0.001 <0.001** |
| LNR | 0.22 (0.15-0.31) | **<0.001** | 0.29 (0.20-0.42) | **<0.001** | 0.23 (0.16-0.34) | **<0.001** | 0.32 (0.21-0.47) | **<0.001** | 0.21 (0.14-0.29) | **<0.001** | 0.28 (0.19-0.40) | **<0.001** |
| PD performed in high volume center  (≥40 PD/year) | 0.87 (0.76-1.01) | **0.077** |  |  | 1.14 (0.96-1.35) | **0.125** |  |  | 0.85 (0.73-0.98) | **0.025** | 0.83 (0.72-0.97) | **0.020** |
| Completeness of pathology report^e^ | 0.77 (0.70-0.84) | **<0.001** | 0.76 (0.69-0.83) | **<0.001** | 1.05 (0.95-1.17) | 0.335 |  |  | 0.79 (0.72-0.87) | **<0.001** | 0.79 (0.72-0.88) | **<0.001** |

**Supplementary table 4.** Predictors for overall survival in 2955 patients after pancreatoduodenectomy for pancreatic cancer using different definitions.

|  | **According to 1mm tumor clearance**  **(main study definition)** | | | | **According to 0mm tumor clearance**  **(secondary definition)** | | | | **According to 1mm tumor clearance,  including anterior surface**  **(secondary definition)** | | | |
| --- | --- | --- | --- | --- | --- | --- | --- | --- | --- | --- | --- | --- |
|  | Univariable analysis  HR (95%CI) | P-value | Multivariable analysis^a^ HR (95% CI) | P-value | Univariable analysis   HR (95% CI) | P-value | Multivariable analysis^b^  HR (95% CI) | P-value | Univariable analysis   HR (95% CI) | P-value | Multivariable analysis^c^  HR (95% CI) | P-value |
| R0 resection^d^ | 0.68 (0.63-0.74) | **<0.001** | 0.72 (0.66-0.79) | **<0.001** | 0.60 (0.55-0.66) | **<0.001** | 0.79 (0.63-0.76) | **<0.001** | 0.67 (0.61-0.72) | **<0.001** | 0.71 (0.65-0.77) | **<0.001** |
| Age | 1.01 (1.01-1.02) | **<0.001** | 1.00 (1.00-1.01) | **0.047** | Same as 1mm tumor clearance | |  |  | Same as 1mm tumor clearance | |  | **0.039** |
| Female | 0.98 (0.90-1.06) | **0.548** |  |  |  |  |  |  |  |  |  |  |
| Chemo(radio)therapy  None  Neo-adjuvant  Adjuvant  Both | reference 0.81 (0.62-1.05) 0.67 (0.61-0.73) 0.52 (0.41-0.67) | **0.104 <0.001 <0.001** | reference 1.03 (0.79-1.35) 0.61 (0.56-0.67) 0.73 (0.57-0.95) | 0.810  **<0.001 0.017** |  |  | reference 1.05 (0.81-1.36) 0.61 (0.56-0.67) 0.70 (0.54-0.90) | 0.718 **<0.001 0.006** |  |  | reference 1.04 (0.80-1.35) 0.61 (0.56-0.67) 0.74 (0.58-0.96) | 0.777 **<0.001 0.022** |
| T stage^e^  1  2  3  4 | reference 1.76 (1.50-2.07) 2.24 (1.89-2.66) 2.35 (1.87-2.95) | **<0.001 <0.001 <0.001** | reference 1.52 (1.29-1.79) 1.82 (1.52-2.17) 1.59 (1.25-2.01) | **<0.001 <0.001 <0.001** |  |  | reference 1.57 (1.33-1.85) 1.83 (1.53-2.19) 1.56 (1.23-1.98) | **<0.001 <0.001 <0.001** |  |  | reference 1.51 (1.28-1.78) 1.81 (1.51-2.16) 1.58 (1.24-2.00) | **<0.001 <0.001 <0.001** |
| LNR | 4.66 (3.93-5.54) | **<0.001** | 4.09 (3.41-4.90) | **<0.001** |  |  | 4.13 (3.45-4.94) | **<0.001** |  |  | 4.08 (3.41-4.89) | **<0.001** |
| PD performed in center with volume ≥40 PD/year | 0.81 (0.74-0.87) | **<0.001** | 0.84 (0.77-0.91) | **<0.001** |  |  | 0.86 (0.79-0.94) | **<0.001** |  |  | 0.84 (0.77-0.91) | **<0.001** |
| Year of resection | 0.98 (0.63-0.74) | **0.006** |  |  |  |  |  |  |  |  |  |  |

Bold numbers in univariable analysis indicates variables that were entered in multivariable analysis. Bold numbers in multivariable analysis indicates statistical significance (p<0.05). OR: Odds ratio. LNR: lymph node ratio. PD: pancreatoduodenectomy. ^a,b,c^Multivariable analysis after backward step selection in 2784 patients (171 deleted due to missing values). ^d^Following the definitions above / on top of the table. ^e^If pT stage was unavailable, cT stage was used.

**Supplementary table 5.** Predictors for overall survival in the subgroup of 214 patients after neoadjuvant treatment using different definitions

|  | **According to 1mm tumor clearance**  **(main study definition)** | | | | **According to 0mm tumor clearance**  **(secondary definition)** | | | | **According to 1mm tumor clearance,  including anterior surface**  **(secondary definition)** | | | |
| --- | --- | --- | --- | --- | --- | --- | --- | --- | --- | --- | --- | --- |
|  | Univariable analysis  HR (95%CI) | P-value | Multivariable analysis^a^ HR (95% CI) | P-value | Univariable analysis   HR (95% CI) | P-value | Multivariable analysis^b^  HR (95% CI) | P-value | Univariable analysis   HR (95% CI) | P-value | Multivariable analysis^c^  HR (95% CI) | P-value |
| R0 resection^d^ | 0.52 (0.36-0.74) | **<0.001** | 0.61 (0.42-0.87) | **0.007** | 0.73 (0.47-1.14) | **0.169** | 0.84 (0.53-1.34) | **0.473** | 0.51 (0.36-0.73) | **<0.001** | 0.60 (0.41-0.86) | **0.006** |
| Age | 1.02 (1-1.04) | 0.124 |  |  | Same as 1mm tumor clearance | |  |  | Same as 1mm tumor clearance | |  |  |
| Female | 0.87 (0.61-1.23) | 0.430 |  |  |  |  |  |  |  |  |  |  |
| Adjuvant chemotherapy | 0.65 (0.46-0.93) | **0.018** | 0.68 (0.47-0.97) | **0.033** |  |  | 0.62 (0.44-0.89) | **0.009** |  |  | 0.68 (0.48-0.98) | **0.037** |
| ASA  1-2  3-4 | reference 0.99 (0.62-1.57) | 0.953 |  |  |  |  |  |  |  |  |  |  |
| Minimally invasive | 1.36 (0.75-2.47) | 0.308 |  |  |  |  |  |  |  |  |  |  |
| T stage^e^  1  2  3  4 | reference 1.22 (0.78-1.90) 2.14 (1.10-4.18) 2.43 (0.93-6.35) | **0.378 0.025 0.069** |  |  |  |  |  |  |  |  |  |  |
| LNR | 8.99 (3.33-24.29) | **<0.001** | 8.29 (2.93-23.47) | **<0.001** |  |  | 110.52 (3.82-29.02) | **<0.001** |  |  | 8.28 (2.93-23.41) | **<0.001** |
| PD performed in center with volume ≥40 PD/year | 1.06 (0.68-1.64) | 0.800 |  |  |  |  |  |  |  |  |  |  |
| Year of resection | 1 (0.91-1.09) | 0.944 |  |  |  |  |  |  |  |  |  |  |

Bold numbers in univariable analysis indicates variables that were entered in multivariable analysis (p<0.20). Bold numbers in multivariable analysis indicates statistical significance (p<0.05). OR: Odds ratio. LNR: lymph node ratio. PD: pancreatoduodenectomy. ^a,b,c^Multivariable analysis after backward step selection in 212 patients (2 deleted due to missing values). ^d^Following the definitions above / on top of the table. ^e^If pT stage was unavailable, cT stage was used.

**Supplementary Figure 1.** Completeness of pathology reporting of pancreatoduodenectomy for pancreatic cancer during

five time periods based on 6 key-items.


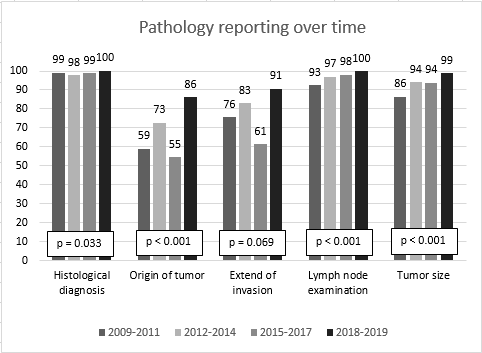

Numbers evaluate the percentage of the pathology reports that described the individual item.
P values describe the increase the increase over time (using linear regression models).
